# Methicillin sensitive *Staphylococcus aureus *producing Panton-Valentine leukocidin toxin in Trinidad & Tobago: a case report

**DOI:** 10.1186/1752-1947-5-157

**Published:** 2011-04-20

**Authors:** Patrick E Akpaka, Stefan Monecke, William H Swanston, AV Chalapathi Rao, Renee Schulz, Paul N Levett

**Affiliations:** 1 Microbiology/Pathology Unit, Department of Para-Clinical Sciences, Faculty of Medical Sciences, University of the West Indies, St Augustine, Trinidad; 2 Institute for Medical Microbiology and Hygiene, Carl Gustav Carus Faculty of Medicine, Technical University of Dresden, Fetscherstrasse 74, D-01307 Dresden, Germany; 3 Eric Williams Medical Sciences Complex, North Central Regional Health Authority, Uriah Butler Highway, Champs Fleurs, Trinidad; 4 Saskatchewan Disease Control Laboratory, 5 Research Drive, Regina, Saskatchewan, S4S 0A4, Canada

## Abstract

**Introduction:**

Certain *Staphylococcus aureus *strains produce Panton-Valentine leukocidin, a toxin that lyses white blood cells causing extensive tissue necrosis and chronic, recurrent or severe infection. This report documents a confirmed case of methicillin-sensitive *Staphylococcus aureus *strain harboring Panton-Valentine leukocidin genes from Trinidad and Tobago. To the best of our knowledge, this is the first time that such a case has been identified and reported from this country.

**Case presentation:**

A 13-year-old Trinidadian boy of African descent presented with upper respiratory symptoms and gastroenteritis-like syptoms. About two weeks later he was re-admitted to our hospital complaining of pain and weakness affecting his left leg, where he had received an intramuscular injection of an anti-emetic drug. He deteriorated and developed septic arthritis, necrotizing fasciitis and septic shock with acute respiratory distress syndrome, leading to death within 48 hours of admission despite intensive care treatment. The infection was caused by *S. aureus*. Bacterial isolates from specimens recovered from our patient before and after his death were analyzed using microarray DNA analysis and *spa *typing, and the results revealed that the *S. aureus *isolates belonged to clonal complex 8, were methicillin-susceptible and positive for Panton-Valentine leukocidin. An autopsy revealed multi-organ failure and histological tissue stains of several organs were also performed and showed involvement of his lungs, liver, kidneys and thymus, which showed Hassal's corpuscles.

**Conclusion:**

Rapid identification of Panton-Valentine leukocidin in methicillin-sensitive *S. aureus *isolates causing severe infections is necessary so as not to miss their potentially devastating consequences. Early feedback from the clinical laboratories is crucial.

## Introduction

*Staphylococcus aureus *has a variety of different virulence factors. Among these, there are hemolysins and leukocidins [1]. A minority of *S. aureus *strains carry bi-component leukocidin. Its genes, *lukS-PV *and *lukF-PV*, are encoded on prophages and can be found in diverse genetic lineages of *S. aureus*. This toxin lyses white blood cells, causing extensive tissue necrosis and severe infection. Strains which are positive for this leukocidin are usually associated with community-acquired infections which generally affect previously healthy children and young adults. It was first described in 1932 by Panton and Valentine [2] and is therefore known as Panton-Valentine leukocidin, or PVL.

Recently the issue of the emergence of novel, community-acquired methicillin-resistant *S. aureus *(MRSA) strains being positive for PVL has been emphasized. However, PVL is also common in methicillin-susceptible *S. aureus *(MSSA) and can be detected in as much as 30% of abscess isolates [3]. In MSSA, it is frequently not diagnosed as there are no phenotypic features or rapid, non-molecular assays available. For that reason, clinical isolates from cases with suspected PVL-associated disease (chronic, recurrent or unusually severe skin and soft tissue infections, necrotizing pneumonia or fasciitis) may further be analyzed using rapid molecular tools whether they were MRSA or MSSA.

There are no previously documented cases of infection by *S. aureus *producing PVL in Trinidad and Tobago and the Caribbean regions. Although the prevalence of MRSA have been reported in Trinidad and Tobago [4], there has never been any report of *S. aureus *carrying PVL genes in this country. We describe here the first confirmed case from Trinidad and Tobago, or, in fact, from any English speaking Caribbean island, of a fatal multi-organ failure caused by a PVL-producing MSSA infection in a previously healthy child. This report stresses the fact that invasive infections due to MSSA could have innocuous symptoms, should not be treated lightly since such infections may have a high mortality rate, and that PVL in MSSA still remains a clinically important issue.

## Case presentation

This is a report of a previously active and healthy 13-year-old Trinidadian boy of African descent with no past medical history, significant history of trauma or travel abroad. He suddenly presented with flu-like symptoms, vomiting and diarrhea of four days duration at a community health center. There was no history of known contact with *S. aureus *infection either at school or with family. He was assessed as a case of viral illness, possibly gastroenteritis, and was treated symptomatically with anti-emetic and analgesic intramuscular injections. Laboratory tests were not pursued and he was discharged with instructions for home care and, if necessary, oral rehydration therapy. About two weeks later he was admitted to the hospital complaining of fever, increasing pain, weakness and inability to lift or to move his left leg where he received an intramuscular injection of the anti-emetic drug.

On admission, his physical examination revealed tender and warm erythematous swelling of his left thigh extending to his upper thigh and hip joint. An ultrasound scan of his left hip, a chest X-ray, electrocardiography and Doppler ultrasound of his popliteal pulses detected no abnormality. Blood cultures, samples of pus from a skin rash and samples for clinical chemistry were taken. Initial laboratory results are given in Table 1.

**Table 1:** Laboratory clinical chemistry results of a fatal case of methicillin-sensitive *Staphylococcus aureus *producing PVL gene in Trinidad and Tobago

Indices	Referral Center	On admission	Normal values
WBC	3.2	1.5	4-11 × 10^9^/L
Hemoglobin	11.2	2.3	11.5 -13.5 g/dL
HCT	30.7	23.8	40-45%
ESR	NT	89	0-15 mm/L
Platelet	148	81	150 -400 × 10^9^/L
Calcium	7.6	7.5	8.4-11.5 mg/dL
Chloride	103	110	92-118 mmol/L
Creatinine	0.9	1.9	0.2- 1.7 mg/dL
CRP	15	90	0-10 mg/dL
Potassium	3.8	7	3.6-5.8 mmol/L
Sodium	138	140	130-148 mmol/L
BUN	13	40	4-24 mg/dL
Uric acid	5.6	13	2.5- 9.0 mg/dL
Phosphorus	4.3	15.1	2.5- 6.5 mg/dL
ALT	34	1211	4- 48U/L
ALP	215	241	38-151U/L
Total Protein	4.9	4.4	6.3-8.6 g/dL

WBC = white cell count; HCT = hematocrit %; ESR = erythrocyte segmentation rate; NT = not tested; CRP = C-Reactive Protein; BUN = Blood Urea Nitrogen; ALT = Alanine transaminases, ALP = Alkaline phosphatase

On further review, the child was assessed as having septic arthritis with a high suspicion of necrotizing fasciitis and septicemia or infective endocarditis. Thus, treatment with clindamycin, ceftriaxone, vancomycin and cloxacillin was started. Later the same day, the cellulitic area around his right knee was noticed to increase rapidly and a computed tomography scan revealed a collection or abscess around his left hip but not involving the capsule of the joint. An immediate exploratory laparotomy and drainage of the pelvic wall abscess under general anesthesia was arranged. During the operation, 200 ml of straw colored fluid was collected and a deep pelvic wall abscess was found, measuring 8 × 6 × 4 cm, adjoining his hip joint capsule and near to the obturator canal. There was thick shiny brown pus in the cavity extending superiorly towards the inlet of his iliac bone, inferiorly to the superior and inferior ramus of his left pelvic bone. The thick joint capsule was intact and there was no evidence of gluteal abscess, but there was a compression from the external and greater tuberosity of the hip bone by the thick pus collection. The pus was drained. Our patient was transferred to the intensive care unit (ICU) although the post-operative condition was very satisfactory. While in the ICU, our patient started to have persistent cough productive of white sputum and was observed to have bilateral crepitations in all his lung fields. A chest X-ray was suggestive of acute respiratory distress syndrome with ground glass appearance. He required inotropes, and had difficulty ventilating resulting in the need for intubation and artificial ventilation. However, our patient's condition deteriorated rapidly and he died 48 hours after admission. An autopsy was remarkable for necrotizing multi-organ failure involving his lungs, kidneys, thymus and other organs. It also revealed congestion, edema and hemorrhage of his lung alveoli, necrosis of his kidney epithelia and Hassall's corpuscles and microabscesses of his thymus gland.

Laboratory results received after the death of our patient revealed grossly abnormal data. These are also shown on Table 1.

## Microbiological diagnosis and molecular analysis of bacterial isolates

Blood, pus and post-mortem specimens yielded growth of *S. aureus *as identified by Gram stain, catalase and coagulase reactions and by biochemistry (MicroScan Walk Away 96 SI, Siemens). The isolates were susceptible to several antibiotics including oxacillin as shown on Table 2. Swab materials from the autopsy also yielded *S. aureus *with same anti-microbial susceptibility pattern. Blood cultures also yielded *S. aureus*.

**Table 2:** Antimicrobial susceptibility test results of a methicillin sensitive *Staphylococcus aureus *producing PVL gene in Trinidad and Tobago

Drug	MIC (μ/mL)	Interpretation
Ampicillin	> 8	R
Amoxycilin/Clavulanic	< 4/2	S
Cefazolin	< 2	S
Ciprofloxacin	< 1	S
Clindamycin	< 1	S
Erythromycin	> 4	R
Gentamicin	> 8	R
Imipenem	< 1	S
Levofloxacin	< 2	S
Linezolid	< 4	S
Oxacillin	< 0.25	S
Penicillin	> 8	R
Piperacillin/Tazobactam	< 1	S
Rifampin	< 1	S
Synercid	0.5	S
Tetracycline	< 4	S
Trimethoprim/Sulfamethoxazole	< 2/38	S
Vancomycin	< 2	S

S = susceptible; R = resistant

The isolates from blood specimens and from swab and tissue specimens at autopsy were further analyzed using *spa *typing [5] and microarray analysis [6]. This allowed us to detect virulence- and resistance-associated genes as well as to assign the isolates to clonal complexes (CC).

The two genotyped isolates were identical and their overall hybridization profile allowed assignation to CC8. Species markers or regulatory genes, including 23S-rRNA gene, *kat*A (encoding catalase), *co*A (coagulase) and *spa *(Protein A) were all positive. The isolates did not harbor *mecA *nor did any other genes associated with staphylococcal chromosomal cassette *mec *elements. Genes *bla*Z (beta lactamase) and associated regulatory genes *bla*I and *bla*R as well as *fos*B (putative resistance marker for fosfomycin, bleomycin) were detected. The isolates carried the hemolysin gamma locus (*lukF, lukS, hlgA*) as well as the genes encoding PVL (*lukF-PV, lukS-PV*). The enterotoxin genes *ent*D, *ent*J, *en*tK, *ent*Q and *ent*R (*sed, sej, sek, seq, ser*) were found, but other enterotoxin genes were not present. The isolates belonged to *agr *group I and capsule type 5. Genes encoding adhesion factors (microbial surface components recognizing adhesive matrix molecules) such as bone sialoprotein-binding protein (*bbp*), clumping factor A and B (*clf*A and *clf*B), cell-wall associated fibronectin-binding protein (*ebh*), immune invasion genes *isa*B (immunodominant antigen B), *isd*A (heme/transferin-binding protein), *lmr*P (putative transporter protein) and genes encoding staphylococcal superantigen-like proteins were also present in these isolates, and their allelic variants were in accordance to CC8 affiliation.

The *spa *typing analysis revealed the isolate to be *spa *type t400. This is quite an uncommon *spa *type, and has only previously been reported from northern Europe [7]. It has also been observed in a PVL-negative mutant of the MRSA strain USA300 (P N Levett, personal communication). Since this strain also belongs to CC8, this confirms the assignment of our isolates to that complex. The repeat pattern of *spa *type t400 (11-19-12-21-17-34-34-22-25) is related to other clonal complex 8 *spa *types (such as t008, 11-19-12-21-17-34-24-34-22-25 or t009, 11-12-21-17-34-24-34-22-24-34-22-33-25).

## Discussion

This case showed an unusually severe clinical presentation. This is similar to previous reports on PVL-producing *S. aureus *[8] causing conditions such as necrotizing pyogenic skin infections, cellulitis, tissue necrosis, septic arthritis, bacteremia, purpura fulminans (typically characterized by disseminated intravascular coagulation and purpuric skin lesions) and community-acquired necrotizing pneumonia.

PVL disease often can be observed in young and healthy people without previous medical history, who might be in close contact to others due to accommodation in barracks or dormitories, or who might be engaged in close contact sport. These risk factors are conceivable in a school child. PVL-positive *S. aureus *have also been transmitted by contaminated articles like sharing towels, razors, poor hand hygiene or illicit drug use. In our case, a transmission by intramuscular injection appears possible, but cannot be proven retrospectively.

The causative strain belonged to CC8. It lacked some of the most prevalent enterotoxins (*egc*-cluster) as well as exfoliative toxins (*et*A, *et*B or *et*D) and epidermal cell differentiation inhibitors (*edin*A, *edin*B or *edin*C). On the other hand, it carried, beside PVL, several different enterotoxin genes (*sed, sej, ser, seb, seq*). The clinical role of these toxins, a possible impact on the virulence, and possible synergistic effects are not yet understood. Thus it cannot be determined how much they contributed to the fatal course of the disease in addition to the PVL. PVL alone is a potent virulence factor, especially with regard to skin and/or soft tissue infections and pneumonia. While enterotoxin genes *sed, sej *and *ser *are common in that clonal complex [8], PVL appears to be rare among CC8-MSSA. The majority of PVL-MSSA infections from geographic areas other than Trinidad and Tobago can be attributed to other clonal complexes, such as CC1, CC5, CC22, CC30, CC80, CC121 and CC152 [3,10]. This could suggest geographic differences in the molecular epidemiology of the PVL-producing MSSA. While PVL genes are uncommon in CC8-MSSA, there is a common and widespread MRSA strain from the same lineage, which is known as USA300. Interestingly, this strain has also been described in Colombia [11], geographically close to Trinidad and Tobago. Thus it is tempting to speculate on a possible phylogenetic relationship between USA300 and the strain described in this study. Further investigations on PVL-positive *S. aureus *in the southern Caribbean and South America are warranted.

Unfortunately, some symptoms, including the hypotension, tachycardia, leukocytopenia, and abnormal liver function tests suggestive of shock, were not adequately addressed until our patient was admitted into the ICU. Some laboratory reports arrived at the ICU only after the death of our patient. Information on the presence of PVL was also obtained only after our patient died. Such problems commonly plague health care providers in the developing world where the facilities and technologies are often not readily available.

The autopsy findings and histological reports proved the involvement of the lungs and consequently their failure. Except for the vague history of flu-like symptoms and the persistent productive cough of whitish sputum noted during the last few hours of his life, there were no major clinical features that suggested pneumonia. An involvement of the kidneys was also noted in this patient in the autopsy findings. These findings emphasize the need for aggressive management of cases of infections by PVL producing *S. aureus *organisms since it appears that no organ or tissues can be spared.

By both phenotypical and molecular methods, it was shown that this strain was susceptible to several relevant antibiotics. A combination of a bactericidal drug, such as a beta-lactam, plus a compound that reduces toxin synthesis, such as clindamycin or rifampicin, is strongly advocated since beta-lactams alone have *in vitro *been shown to increase PVL in synthesis studies [12]. However, susceptibility tests need to be performed urgently in order to assess the efficiency of the therapy and to rule out PVL-MRSA. Thus, the initial choice of antibiotics in the presented case appeared to be correct, but nevertheless the case resulted in a fatal outcome. This emphasizes the severity of PVL-associated disease.

## Conclusion

Given the ability of PVL-producing *S. aureus *(either MSSA or MRSA) to cause life-threatening disease, and the absence of any rapid non-molecular tests for PVL, the crucial role of awareness cannot be over-emphasized. This report provides timely and informative hints to all health care facilities, on a local or regional level, that clinical presentation of PVL-producing *S. aureus *infections should not be underestimated. It is also the first report of a confirmed case of PVL-producing *S. aureus *in Trinidad and Tobago and in the English speaking Caribbean islands.

## Consent

A written informed consent was obtained from the patient's next-of-kin for the publication of this case report and any accompanying images. A copy of the written consent is available for review by the Editor-in-Chief of this journal.

## Competing interests

The authors declare that they have no competing interests.

## Authors' contributions

PEA and WHS carried out the clinical study of the patient. AVCR carried out the autopsy and histological staining. SM, RS, PNL performed the molecular analyses. PEA drafted the manuscript. All authors read and approved the final manuscript.
